# ‘The End of Sitting’ in a public space: observations of spontaneous visitors

**DOI:** 10.1186/s12889-017-4971-7

**Published:** 2017-12-08

**Authors:** Lidewij R. Renaud, Maaike A. Huysmans, Erwin M. Speklé, Allard J. van der Beek, Hidde P. van der Ploeg

**Affiliations:** 1 Department of Public and Occupational Health and Amsterdam Public Health research institute, VU University Medical Center, Van der Boechorststraat 7, 1081 BT Amsterdam, The Netherlands; 2 Arbo Unie OHS, Diakenhuisweg 25, 2033 AP Haarlem, The Netherlands

**Keywords:** Sitting, Office, Landscape, Visitors, Upright

## Abstract

**Background:**

Sitting too much has been associated with negative health outcomes. *‘*The End of Sitting’ is a newly developed office landscape that moves away from the traditional chair-desk setup. The landscape aims to reduce sitting time by offering a variety of (supported) standing positions. The aim of this study was to determine the usage of the landscape after being placed in the main entrance hall of the VU University in Amsterdam.

**Methods:**

We observed the number of spontaneous visitors as well as the duration of visits, changes to another location within the landscape, and adopted postures. Using questionnaires reasons (not) to visit the landscape, perceived affordances of the landscape and associations with long-term use were determined.

**Results:**

Observed numbers of visitors were relatively low and duration of visits were short, which seemed to indicate visitors were trying out the landscape. The majority of visitors were in an upright position, reflecting the designers’ intentions. Visitors indicated that long-term use would be pleasant to them.

**Conclusion:**

‘The End of Sitting’ landscape received positive reactions but number of visits were limited in the few months that it was placed in the university main entrance hall. The landscape might be better suited for designated working or study spaces, for which it was originally intended. It might also be worth to explore the landscapes suitability for short stay environments, such as waiting rooms.

**Electronic supplementary material:**

The online version of this article (10.1186/s12889-017-4971-7) contains supplementary material, which is available to authorized users.

## Background

The health risks of high levels of sitting time are well documented and have been associated with the development of diabetes, cardiovascular diseases and premature mortality [1–3]. It seems that these effects can only be marginally attenuated by physical activity [3] and there is a need for strategies to reduce sedentary behaviour [4, 5].

Since office workers spend a large part of their workday seated [6], the workplace is a highly conductive environment for health promotion [7]. Several interventions to reduce sitting time have been developed for office workers [8–10]. In these interventions the aim is to reduce chair use and subsequently reduce sitting time, increasing movement or standing time. Furthermore, standing time has been inversely associated with all-cause mortality, and has been identified as a promising alternative for sitting in the workplace [4, 11, 12].

Inspired by the research around the health risks of sitting time, RAAAF (Rietveld Architecture Art Affordances) and visual artist Barbara Visser created an office landscape of the future called ‘The End of Sitting’, completely abandoning the conventional setting of office chair and desk. A detailed description of the development of and the theory behind the landscape can be found elsewhere [13]. In short: as an office chair affords sitting, the developed landscape affords a variety of (supported) standing positions. The landscape was designed to stimulate visitors to frequently change their posture and location in the landscape.

Withagen & Caljouw used a lab setting to compare ‘The End of Sitting’ to a conventional office, by studying 18 subjects who performed a semi-standardized task (preparing a presentation within 75 min) in both environments [14]. They found that most of the subjects (83%) worked in more than one non-sitting posture in the landscape, whereas all but one of the subjects worked in a sitting posture in the conventional office. Also subjects felt more energetic, although they experienced higher levels of fatigue in the legs after working in the landscape. To date, it has not been investigated how the landscape would be used in a real world setting.

In the current study, we observed whether this new environment would attract spontaneous visitors, when placed in an area of the University main entrance hall that is generally used as transit, study, meeting and break area. Hence, our primary research question was: How many people does the landscape attract? Additionally, we addressed the following secondary questions: What is the duration of the visits? Do visitors change location within the landscape and which postures and activities do visitors adopt? Finally, we examined reasons (not) to visit the landscape, perceived affordances of the landscape, and perceived associations with long-term use.

## Methods

### The landscape and study design

**Fig. 1:**
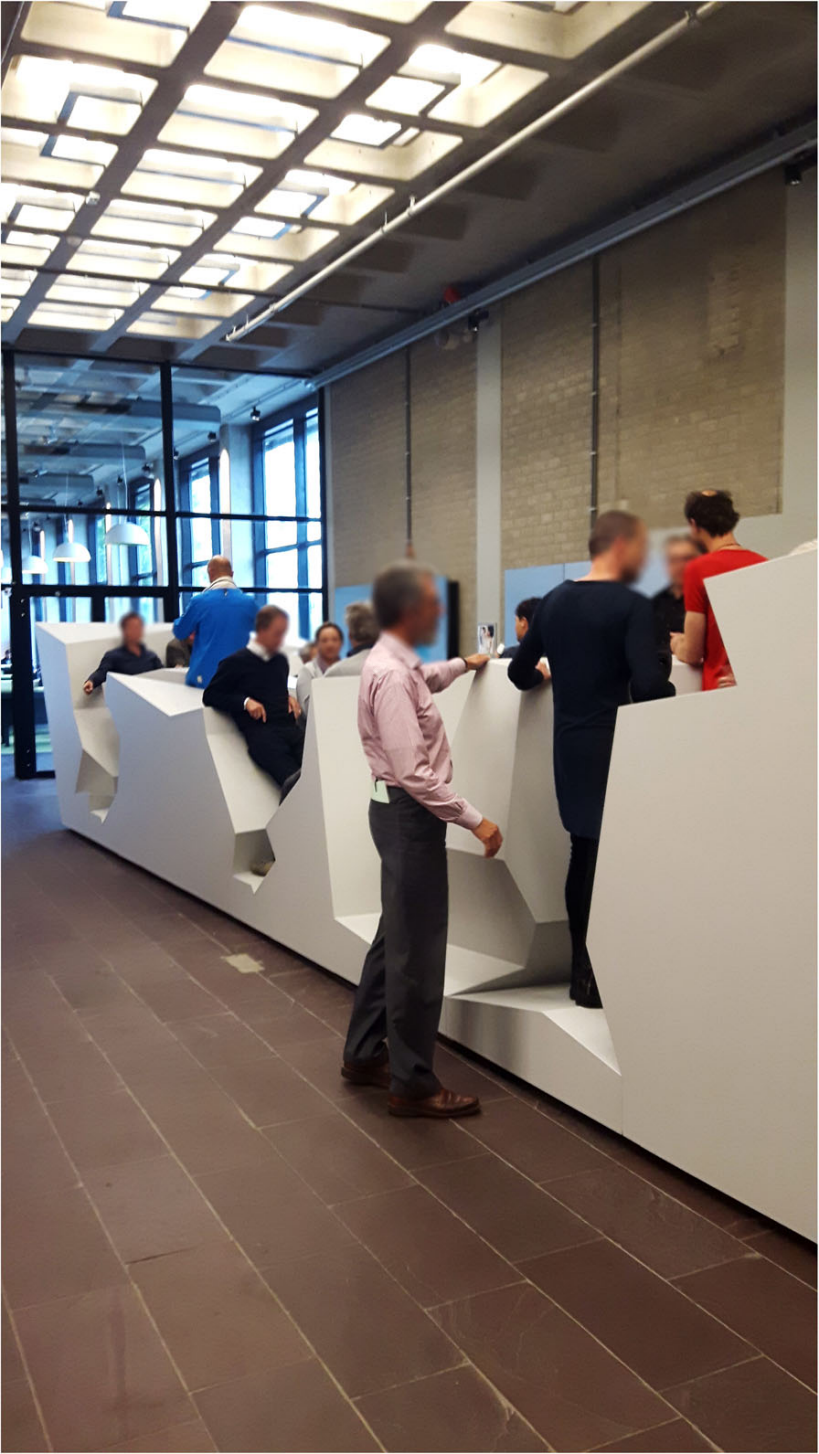
Cut-out of The End of Sitting placed in the main entrance hall of the university during a meeting with the purpose to try out the landscape

A cut-out of the original office landscape was placed in the main entrance hall of the VU University Amsterdam in the Netherlands. The landscape – a 12 × 3 meter cut-out of the original ‘End of Sitting’ landscape – was placed in the main entrance hall of the VU University from March–May 2016 (Fig. 1). The first month, the landscape drew some local and national media interest. No measurements were performed during this first period. An A3 sized poster with background information about the health risks of prolonged sitting and the development of the landscape was attached to the side of the landscape, to inform (potential) visitors. The poster also stated that the landscape was free to be used. No further prompts to attract visitors to the landscape were provided, resulting in solely spontaneous visitors. To answer the research questions, three different research methods were used: continuous observations, observational scans, and questionnaires. Data were collected during April and May 2016. The Medical Ethical Committee of the VU University Medical Center in Amsterdam approved the study.

### Continuous observations

Continuous observations were performed to assess number of visitors, duration of visits and changes in location within the landscape. These observations were added to the research method because the observational scans (described below) provided too little information on short term visitors, which were the most prevalent visitors. The researcher recorded the start and end time of every visit on the landscape. Visiting times were rounded up to full minutes (e.g. a duration of 1 min indicates the visit lasted 1 min or less). Furthermore, the researcher recorded whether the visitor changed location to at least one other location within the landscape (dichotomously). Also, gender and estimated age group (child, student, other adult, senior) were recorded in a logbook. The continuous observations took place between 11.50 h and 14.20 h during 6 week days, resulting in a total of 15 h of observations. When visitors left the landscape, they were asked by a second researcher to complete a questionnaire for landscape visitors.

### Observational scans

**Fig. 2:**
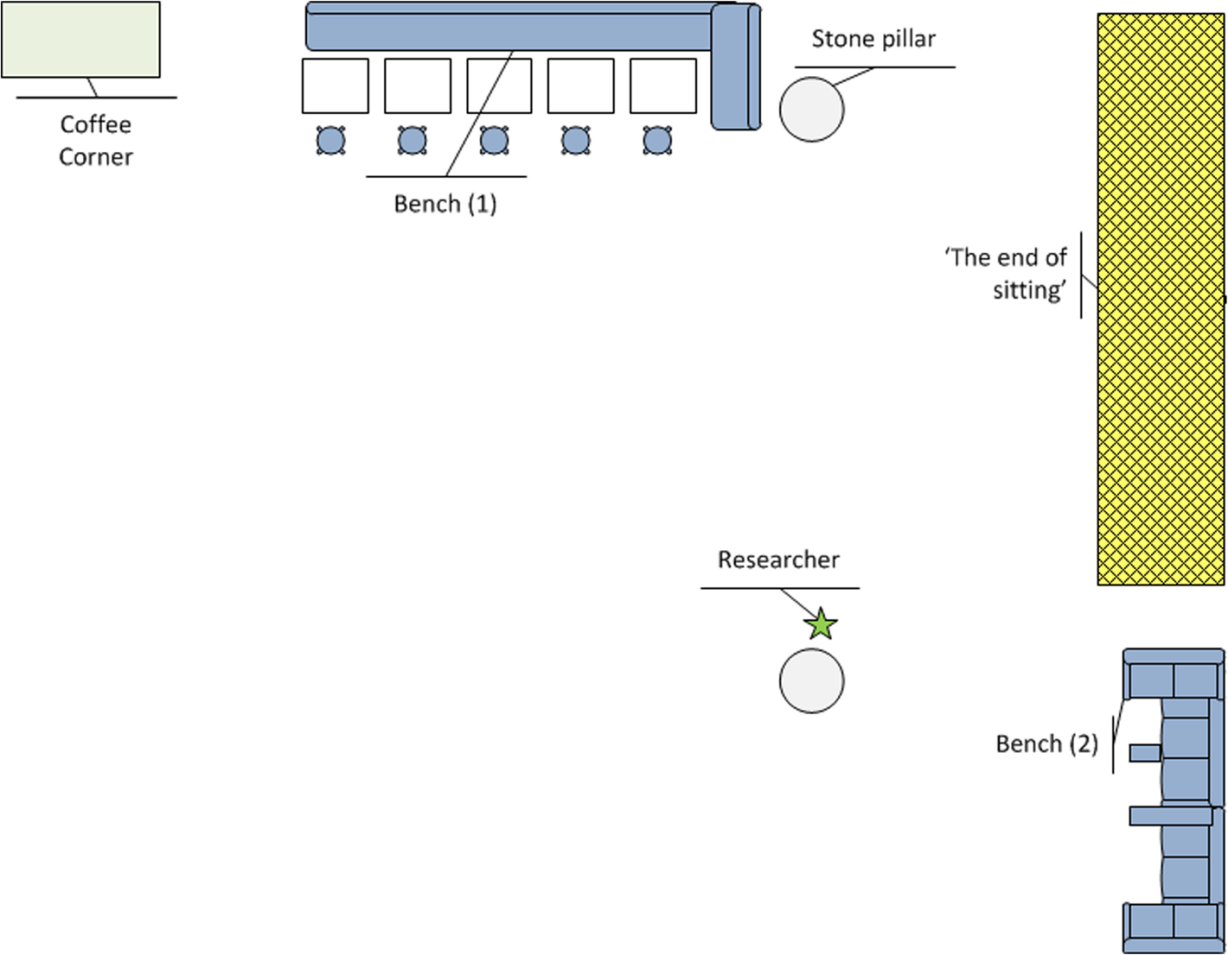
Research area with benches (1 and 2), the landscape ‘The End of Sitting’ and the observation point from where the researchers conducted their observations

Observational scans were used to assess number of visitors and adopted postures and activities. In accordance with the SOPARC and SOPLAY methods [15, 16], an observation method was developed, piloted and optimized. To compare number of visitors, adopted postures and activities, we included spontaneous visitors of the surrounding benches, which were already present in the main hall before the installation of the landscape (see Fig. 2 for an overview of the study area). During the scan the study area (landscape + benches) was observed from left to right, and the following was recorded: presence of individuals in the landscape or the surrounding benches, their body posture (as specified by Withagen & Caljouw [14]; sit, lean, stand, stoop stand, lay back, lay belly, other); their activity (drinking or eating, interaction with a device, reading or writing on paper, interaction with other people, general leisure activity); gender and estimated age group. Additionally, with every scan, the outside temperature (°C) and weather condition (sunny, half cloudy, cloudy or rainy) were recorded.

Observations were made during 8 week days performing six scans per day, three scans every hour starting from 10.50 h and three scans every hour starting from 13.20 h. These times were selected because it was likely that more people would be in the main entrance hall, since it was 10 min before classes would start or it was during lunch or class breaks. This resulted in a total of 48 scans, performed by the researcher or a research student. After every observational scan, a questionnaire for bench and landscape visitors was distributed from left to right in the bench area and the landscape, respectively. This could mean that visitors who had been observed during the scan, did not always complete the questionnaire because they might have left before the distribution of the questionnaire, and vice versa.

Interrater reliability for the observational scans was determined by comparing observations of 2 days (12 scans, 98 observed individuals), performed separately by the two researchers simultaneously, 1 day before the measurement period and 1 day during the measurement period.

### Questionnaires

The visitors of the landscape and the benches received different questionnaires, with some overlapping questions. The questionnaires focussed on activities, postures, visiting duration, change of location, reasons (not) to visit the landscape, perceived affordances of the landscape, and associations with long-term use. An overview of questions and answering categories can be found in Additional file 1: Appendix A. In both questionnaires, data on gender, age and type of visitor (student, employee or visitor) were collected. The questionnaires were available in Dutch and English. The number of individuals who declined to complete the questionnaire or had already completed the questionnaire were recorded.

### Statistical analysis

The collected data from the continuous observations, the observational scans and the questionnaires were analysed using IBM SPSS Statistics 22 to generate descriptive statistics and interrater reliability coefficients (Cohen’s Kappa).

## Results

Despite training, the researchers did not find consensus on the postures as proposed by Withagen & Caljauw [14] which was indicated by a Cohen’s Kappa of −0.04. The posture data was reliable when only a distinction was made between a sitting and an upright position (lean, stand or stoop stand) with a Cohen’s Kappa of 0.94. Hence, postures were only assessed as sitting or upright. The interrater reliability for all activities (Cohen’s Kappa of 0.77 and higher) and location (Cohen’s Kappa 0.98) were good.

### Number of visitors and demographics

During the 15 h of continuous observations, 62 landscape visits were observed, which was 4 landscape visits on average per observed hour. During the observational scans 510 visitors were observed, of whom 43 (8.4%) were landscape visitors, which resulted in on average 1 landscape visitor per scan. The majority of the observed bench and landscape visitors were of a student’s age (75.3 and 90.7%, respectively). In total 292 visitors completed a questionnaire, of whom the majority were bench visitors (80.8%) and women (62.5%), the mean age was 26.0 years (sd = 10.6 years). Table 1 provides an overview of numbers of visitors captured by the different measurement methods.

**Table 1:** Number of visitors observed during continuous observations, observational scans and number of visitors who completed a questionnaire

Location	Continuous observation (% males)	Observational scan (% males)	Questionnaires (% males)
Bench	–	467 (31.9)	236 (33.5)
Landscape	62 (53.2)	43 (67.4)	56 (53.5)
Total		510 (34.9)	292 (37.5)

### Duration of visits and change of location

#### Continuous observations of the landscape

The 62 landscape visits recorded during the continuous observations had a median duration of 2 min and the mean duration was 8 min (sd = 15 min and max = 120 min). Twelve (19.4%) landscape visitors changed their location during their visit, of which all but one visit had a duration of 8 min or less.

#### Self-reported duration of visits and change of location

**Fig. 3:**
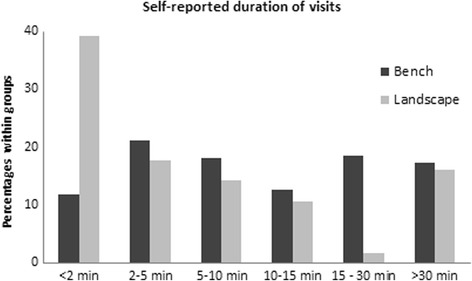
Self-reported duration of bench and landscape visits

In general, self-reported duration of visits to the benches were of more variable length than those of the landscape, which were shorter, with almost 40% of the landscape visits being shorter than 2 min (see Fig. 3).

Among the landscape visitors 66.1% reported they visited the landscape for the first time and 21.4% had visited the landscape once previously (data not shown). Also, 51.8% reported to have visited only one location in the landscape during their current visit, while 23.2%, 21.4%, and 3.6% reported two, three and four or more locations, respectively. Of the nine visitors who reported a duration of visit of >30 min, two reported visiting more than one location during that visit.

### Adopted activities and postures and perceived affordances

#### Observational scans **-** adopted activities and postures

During observational scans, the main activity of bench visitors was interaction with others (66.2%), followed by eating or drinking (49.9%). On the landscape the main activity was interacting with a device (74.4%), directly followed by interaction with others (72.1%) as second activity (see Table 2). During observational scans, 96.4% of the bench visitors was in a sitting position, while 90.7% of the landscape visitors were in an upright position.

**Table 2:** Observed activities during observational scans, self-reported activities and self-reported postures and affordances for the landscape

	*N* (%)^a^	
Observed activities	Benches	Landscape
Eating or drinking	233 (49.9)	10 (23.3)
Interaction device	212 (45.4)	32 (74.4)
Interaction others	309 (66.2)	31 (72.1)
Reading/writing paper	50 (10.7)	3 (7.0)
General leisure	11 (2.4)	0.0
Self-reported activities^b^		
Drinking (Coffee)	80 (33.9)	6 (10.7)
Eating / having lunch	61 (25.8)	3 (5.4)
Interaction with phone	76 (32.2)	9 (16.1)
Using a laptop / tablet	45 (19.1)	10 (17.9)
Chatting with others	109 (46.2)	23 (41.1)
Reading / writing paper	13 (5.5)	5 (8.9)
Waiting for someone	57 (24.2)	11 (19.6)
Other	14 (5.9)	10 (17.8)
Self-reported postures^b^		
Sitting		14 (25.0)
Standing		33 (58.9)
Laying down		4 (7.1)
Leaning against		39 (69.6)
Squatting		6 (10.7)
Affordances ‘This landscape invites to’^b^		
Sit in		13 (23.2)
Stand in		20 (35.6)
Lay down		2 (3.6)
Change posture		17 (30.4)
Climb		13 (23.2)
Change location		7 (12.5)
Lean against		31 (55.4)
Affordances ‘This landscape is most suitable to’^b^		
Study / read		16 (28.6)
Use a phone		18 (32.1)
Have a break		33 (58.9)
Have a meeting		16 (28.6)
Brainstorm		19 (33.9)
Eat / drink		11 (19.6)
Use tablet / laptop		11 (19.6)

^a^In brackets the percentage of within groups are shown

^b^Visitors were able to give multiple answers to this question

#### Self-reported activities and postures and perceived affordances

The self-reported activities by the bench and landscape visitors and postures and perceived affordances for landscape visitors are shown in Table 2. Chatting with others was both on the benches and the landscape the most often reported activity (46.2% and 41.6%, respectively). Drinking (coffee), eating / having lunch and interaction with a phone was more often reported by the bench visitors as compared to the landscape visitors. In total 24 visitors wrote down ‘other activity’, of which 20 were themed as ‘other leisure activities’ and four (landscape visitors) as ‘trying out the landscape’.

Most self-reported postures adopted on the landscape were leaning against (69.6%) and / or standing (58.9%), while 25.0% indicated they (also) sat during their visit. Lean against (55.4%) and stand in (35.6%) were also the most reported postures for which the landscape invites to. Still, in general, visitors did not see an affordance to change location (12.5%). The majority found the landscape most suitable for having a break (58.9%), but to a lesser extent to eat or drink in it (19.6%).

### Reasons (not) to visit the landscape and associations with long-term use

#### Self-reported reason (not) to visit the landscape

Most bench visitors indicated that they ‘prefer sitting on a bench’ (42.8%) or ‘it does not look inviting to me’ (34.7%) as the reason why they did not visit the landscape (see Table 3). There were 37 bench visitors who indicated an ‘other’ reason not to visit the landscape, which included: I do not know its purpose (*N* = 11), I am usually not here (*N* = 11), not interested (*N* = 6), I would feel awkward (*N* = 6), and it is uncomfortable (*N* = 3).

**Table 3:** Reasons to visit the landscape and reasons not to visit the landscape (again)

	*N* (%)
Reason not to visit landscape (bench visitors)^a^	
I prefer sitting on a bench	101(42.8)
It goes against my routine	28 (11.9)
I do not have time for it	21 (8.9)
I did not notice it before	34 (14.4)
I think it is not practical for use	67 (28.4)
It does not look inviting to me	82 (34.7)
I thought it was not allowed	30 (12.7)
Other	37 (15.7)
Reason to visit landscape (previously)^b^	
Curious	46 (49.5)
Following friends/colleagues	19 (20.4)
No place somewhere else	4 (4.3)
Nice place	9 (9.7)
Read about it	5 (5.4)
Other	10 (10.8)
Total	93 (100)

^a^ Visitors were able to tick multiple boxes; percentages represent the quantity of boxes checked within the bench users

^b^ Single answer question, percentages shown are from the total of landscape visitors

In the questionnaire, 37 (15.7%) of the bench visitors indicated to have visited the landscape previously and they also answered the question about their reason to visit, resulting in 93 respondents for this question when added to the landscape visitors who completed a questionnaire. Most visitors indicated they had visited the landscape (previously) because they were curious (49.5%). The 10 ‘other’ reasons to visit the landscape, could all be themed as ‘being a convenient place’. Some visitors marked more than one reason, besides ‘curious’ (data not presented).

#### Perceived associations with long-term use

In Table 4 an overview of associations with perceived long-term use is presented. Most of the landscape visitors indicated that long-term use would be pleasant to them (43.6%) and 37.0% indicated that it would give them energy, while for the other categories most visitors scored ‘neutral’.

**Table 4:** Associations with long-term use of the landscape

Long-term use would…	Agree, *N* (%)	Neutral, *N*(%)	Reverse, *N* (%)
make me more productive	14 (25.9)	29 (53.7)	11 (20.4)
make me more creative	20 (36.4)	30 (54.5)	5 (9.1)
give me energy	20 (37.0)	19 (35.2)	15 (27.8)
be physically easy	17 (31.5)	25 (46.3)	12 (22.2)
make me relaxed	17 (31.5)	27 (50.0)	10 (18.5)
be pleasant to me	24 (43.6)	23 (41.8)	8 (14.5)

## Discussion

This study examined whether ‘The End of Sitting’, when placed in a university main entrance hall would attract spontaneous visitors. During the continuous observations and the observational scans 4 visitors per hour and 1 visitor per scan were observed, respectively. Together with the low observed (median = 2 min) and self-reported (39.3% <2 min) duration of visits, this shows that visits to the landscape were sporadic and mostly of short duration. Almost half of the visitors reported that they visited the landscape because they were curious.The local and national media coverage of the placement of the landscape and its purpose to reduce sedentary behaviour presumably contributed to increased awareness among potential visitors. Still, the benches surrounding the landscape were used more frequent and many bench visitors reported they preferred sitting on a bench (42.8%) as reason not to visit the landscape. The limited visits to the landscape might have partly been due to the presence of the traditional seating areas nearby. Also, the lack of an accepting culture for such a radical redesign of the seating areas could have been restricting to influence prospective visitors to its full potential. Potential visitors might not have noticed the landscape or understood its purpose and might have overlooked the poster that explained the landscapes purpose. Scarce numbers of visitors have been reported before when introducing an environmental change to decrease sitting time in a public space, as exemplified by a study that introduced desk bikes in common spaces at a Belgium airport and train stations [17]. In an office environment, when introducing alternative workstations next to conventional workstations, office workers also seemed to prefer the sitting options [18].

In line with the developers’ intentions [13], the majority of landscape visitors were in an upright position (90.7%) during the observational scans and self-reported data showed that visitors were mostly leaning against (69.6%) and/or standing (59.9%). Although the landscape was designed to enable changes in posture and location within the landscape, most visitors (80.6%) did not change location. However, self-reported numbers of no change of location were lower (51.8%). Still our observed lack of variation in location might have been due to the short duration of visits. Withagen & Caljauw [14] showed more variation in locations in their laboratorial study, where subjects were in the landscape for 75 min to finish a predefined task. They showed that 44% of the subjects worked on two locations, 17% on three locations, 22% on four locations and only 17% on just one location. Nevertheless, the landscape in which their experiment took place was considerably larger than the current cut out version.

Visitors indicated that long-term use of the landscape would be pleasant to them (43.6%). Beside the earlier described cardio-metabolic health risks of prolonged sitting, uninterrupted standing also has negative health effects [19]. It seems important to alternate sitting, standing and physical activity throughout the day, although there is no consensus on the frequency for varying postures to reduce musculoskeletal risks or long-term health risks [20]. Nevertheless, it has been shown that training can intensify the use and thereby the alternation in postures, of alternative workstations [21, 22].

The majority of landscape visitors (58.9%) found the landscape most suitable for taking a break. This finding about the break affordance of the landscape may have been driven by the specific location in the university main entrance hall, which is not only a transit area but also used for taking a break, meeting and studying. In a typical office environment, performed activities and self-reported possible work task affordances may have been different. Still, creative office-like tasks were reported with 33.0% indicating the landscape was most suitable to brainstorm, substantiated by 36.4% indicating the landscape would make them more creative with long-term use.

To our knowledge, this was the first study on ‘The End of Sitting’ in a real-life setting. We adapted existing observational methods (SOPARK and SOPLAY) to develop a novel method to observe landscape usage, which might also be suitable for future research studies on the built environment. We combined 48 observational scans with 15 h of continuous observations providing an indication of the impact of the landscape, when placed in a public environment. However, numbers of observed visitors and questionnaire respondents for the landscape were lower than expected and hampered statistical power such that no further subgroup analyses could be performed.

Another limitation of the study was the insufficient consensus between the two researchers with regard to scoring the different standing postures, as proposed by Withagen & Caljauw [14]. Hence, for the observed postures only the distinction between sitting and standing could be made. Video analyses might have been more suitable for scoring postures in such a detailed manner, rather than the real time direct observations utilised in this study. Still, due to the ethical aspects of video recording spontaneous visitors, real-time observations had the preference. Furthermore, using the self-reported questionnaire data, we were able to get more detailed data on postures.

A transit area (such as the main entrance hall of the university) might not be the most suitable space for placement. The landscape might be better suited for designated working and studying spaces, for which it was originally intended. When placing the landscape in an office environment, the researchers recommend focusing on assisting potential visitors to adapt to the drastic environmental change. It is recommended to provide additional training on practical use, since both static sitting as well as static standing should be avoided [23]. The landscape could be used performing certain work tasks for which creativity is required, such as brainstorm sessions. It has been proposed that a reduction of comfort levels can increase variation of posture and movement [24], which the landscape might induce when used for longer periods of time than we observed in the current study. Future research should focus on the landscape as part of a real-life office setting in which the landscape could be the only option or it could be placed next to more traditional working areas. In the latter case, not only more attention should be focussed on informing workers of proper usage but also on attempts to change perceptions and culture around landscape usage. A behavioural change program might further facilitate landscape visits, as was shown for sit-stand workstations [21].

It might also be worth to explore the landscapes suitability for short stay environments, such as waiting rooms, replacing all sitting options (except for people with reduced mobility). In this way people would be ‘forced’ to change their regular routine of sitting down while waiting. Future research could focus on the impact of the landscape placed in an area without alternative sitting options, which might facilitate visits and cultural adaptation.

## Conclusion

The placement of the ‘The End of Sitting’ in a public space did attract spontaneous visitors. Although they were not high in numbers and the duration of visits were short, the vast majority of visitors was in an upright position. This landscape is one of the first alternatives for the conventional chair-desk setup and should be seen as an encouragement for intervention developers in the (occupational) health field. However, a transit area (such as the main entrance hall of the university) might not be the most suitable space for placement, at least not with the current culture of preferred sitting. Designated working or study areas as well as waiting areas might be better suited for ‘The End of Sitting’ landscape.

## Additional file

Additional file 1: Appendix A. ‘Questions from bench (B) and landscape (L) questionnaires with answering categories’. (DOCX 15 kb)

## Abbreviations

RAAAF: Rietveld architecture art affordances
