# Supplementary material for: ‘The End of Sitting’ in a public space: observations of spontaneous visitors
Source: BMC Public Health. 2017 Dec 8;17:937. doi: 10.1186/s12889-017-4971-7 (PMC5721657; doi:10.1186/s12889-017-4971-7)
Supplement: Additional file 1: Appendix A. — ‘Questions from bench (B) and landscape (L) questionnaires with answering categories’. (DOCX 15 kb) [file 12889_2017_4971_MOESM1_ESM.docx]

**Additional file 1: Appendix A.** Questions from bench (B) and landscape (L) questionnaires with answering categories.

| **Questions for Bench (B) and/or Landscape (L) visitors** | **Answering categories** |
| --- | --- |
| How long have you been on this bench / landscape now? (B, L) | less than 2 minutes 2 – 5 minutes 5 – 10 minutes 10 – 15 minutes 15 – 30 minutes more than 30 minutes |
| What were you doing during your stay on this bench / the landscape? (B, L) | drinking (coffee)* eating / having lunch using my phone using my laptop / tablet chatting with others reading paper (e.g. magazine, newspaper, etc.) waiting for someone other, namely: |
| What is the reason you did not go on the landscape (this time)? (B) | I prefer sitting on a bench* It goes against my routine I do not have time for it I did not notice it before  I think it is not practical for use It does not look inviting to me I thought it was not allowed other, namely: |
| Have you visited the landscape in the previous weeks? (B) | yes no |
| If yes: what is the reason you went into the landscape earlier? (B) / what is the reason you went into the landscape? (L) | I was curious my friends / colleagues went into it there was no place available elsewhere it is a nice place I read about it in the media / newsletter other, namely: |
| How many different places in the landscape have you visited during you current visit? (L) | 1 2 3 4 more than 4 |
| Which posture(s) did you adopt during your visit? (L) | sitting* standing laying down leaning against squatting |
| Did you previously visit the landscape in the past few weeks? (L) | no yes one time before yes twice before yes three times before yes more than three times before |
| This landscape invites people to (L) | sit in* stand in lay down change body postures climb  change location lean against other, namely: |
| In my opinion, when it comes to work or study related tasks, this landscape is most suitable to: (L) | study / read* use the telephone have a break  have a meeting brainstorm eat / drink  work on a laptop / tablet other, namely: |
| Long term use of the landscape would… (L) | make me more productive (or less productive)#  make me more creative (or less creative)  give me energy (or cost me energy)  be physically easy (or give physical discomfort) make me relaxed (or make me tensed) be pleasant to me (or be unpleasant to me) |

*Multiple answers could be provided.
#Between the two options, the option ‘neutral’ was offered.
